# Molecular Network Analysis in Model and Non-Model Legumes: Challenges in Omics Data Interpretation Across Species, with a Focus on *Glycine max, Lupinus albus* and *Medicago truncatula*

**DOI:** 10.3390/plants14233586

**Published:** 2025-11-24

**Authors:** Nayla Zalzalah, Jakob Bruggink, Mohamad Elian, Simon Lackey, Julia C. Wozny, Siwar Haidar, Elroy R. Cober, Tim Xing, Bahram Samanfar

**Affiliations:** 1 Ottawa Research and Development Centre, Agriculture and Agri-Food Canada, Ottawa, ON K1A 0C6, Canada; 2 Department of Biology, Ottawa Institute of Systems Biology, Carleton University, Ottawa, ON K1S 5B6, Canada

**Keywords:** molecular network analysis, omics data integration, *Glycine max*, *Lupinus albus*, *Medicago truncatula*, non-model plants, co-expression networks, cross-species gene analysis, legumes, orphan crops

## Abstract

Molecular network analysis offers powerful insights for plant improvement by capturing complex regulatory interactions. However, translating omics data across species presents significant challenges. Non-model crops such as soybean and lupin often lack comprehensive genomic resources, which complicates network analysis. Model species (e.g., *Arabidopsis thaliana*) provide rich data but may lack legume-specific pathways. This review synthesizes these challenges and examines legume networks in soybean, lupin, and the model legume, *Medicago truncatula*. Strategies such as multi-omics integration and Artificial Intelligence (AI)-driven tools, combined with wet lab validation studies such as clustered regularly interspaced short palindromic repeats (CRISPR), are discussed to bridge the gap between discovery and application. Ultimately, we conclude that cross-species multi-omics integration, empowered by AI and validated by gene editing, will be pivotal for translating network discoveries into resilient legume crops. Strategic investments in under-researched non-model legumes and advanced molecular tools are essential to ensure sustainable agriculture and future crop resilience.

## 1. Introduction

Understanding molecular networks that control plant growth, development, and stress response is increasingly important for improving crop productivity and resilience [1]. Advances in next-generation sequencing and omics techniques—comprehensive large-scale analyses of DNA (genomics), RNA (transcriptomics), proteins (proteomics), metabolites (metabolomics) and phenotypes (phenomics)—have helped researchers map gene regulatory networks in greater detail. However, success ultimately depends on genomic and functional data quality. Non-model species, including critical global crops such as soybean and emerging legumes such as lupin, have traditionally lagged behind model organisms due to several inherent challenges. Soybeans’ paleopolyploid genome, characterized by extensive gene duplication, complicates accurate annotation and functional assignment of gene families [2]. Lupin, despite its nutritional potential and agronomic benefits, has historically suffered from limited genomic resources, restricting network analysis reliability [3].

Model species such as *Arabidopsis thaliana* benefit from a wealth of community-curated resources like The Arabidopsis Information Resource, (TAIR) [4]. These resources have facilitated detailed regulatory maps and validation through analysis of mutant libraries and transgenic complementation studies [5,6]. Even in well-studied systems like *A. thaliana*, integrating different omics layers into cohesive models remains challenging. Interpreting these dense networks requires advanced computational techniques and often fails to capture dynamic interplay observed in natural or field conditions [7]. Inzé and Nelissen [8] show that while only about 1.3% of regulatory genes discovered in Arabidopsis successfully translate to crops like maize, these insights still provide valuable templates for comparative analysis.

*Medicago truncatula* serves as an intermediate model legume bridging Arabidopsis and crops like soybean (*Glycine max*) and white lupin (*Lupinus albus*). Its compact diploid genome of roughly 465 Mb and its well-characterized symbiosis with rhizobia provide a more relevant reference for legume-specific traits than Arabidopsis. Comparative mapping shows about 75 percent synteny with soybean genes and roughly 70 percent with lupin, versus only about 60 percent with Arabidopsis [3,9]. While Arabidopsis still benefits from the most extensive bioinformatic resources, *M. truncatula* offers a closer phylogenetic framework for translating omics insights across legumes [3,9]. Table 1 provides a comparative overview of these species and their relevance to molecular network analysis. In the following sections, we outline the major challenges in molecular data interpretation for non-model versus model legumes (Section 2). We then explore integrative strategies, notably multi-omics and AI/machine learning tools, aimed at bridging the gap between discovery and application (Section 3). Following this methodological foundation, Section 4 examines omics integration across data layers (genome, transcriptome, proteome, metabolome, and phenome). Section 5 highlights specific examples of cell signaling networks regulating stress responses. Finally, we discuss future research directions for orphan legumes (Section 6) and present our conclusions (Section 7).

## 2. Interpretation Challenges in Molecular Data

Network-based methods are now a key part of how we interpret high-throughput omics data in plants. Instead of focusing on individual genes, these approaches examine patterns of coordinated expression across conditions, allowing researchers to uncover complex regulatory relationships. One of the main outcomes of this type of analysis is the identification of modules, or groups of genes that show similar expression patterns and often share biological functions. Within these modules, hub genes are those with the highest level of connectivity and may play important regulatory [10]. Several frameworks exist for building gene networks, including correlation-based models, Bayesian approaches, and methods that use mutual information (e.g., ARACNE) [11]. Among these, Weighted Gene Co-expression Network Analysis (WGCNA) has become especially widely adopted in crop studies due to its strong statistical foundation and ability to reveal biologically meaningful patterns [10]. In legumes such as soybean and lupin, network analysis has been used to identify modules linked to traits like nodulation, nutrient response, and seed quality [12,13]. Still, translating network patterns into biological meaning is not always straightforward, particularly in non-model systems, where variation in gene function and limited annotation resources can make interpretation more difficult.

### 2.1. Non-Model Legumes (Soybean, Lupin)

Soybean’s paleopolyploid history and incomplete genome annotations mean many genes remain labeled as “hypothetical” or “putative,” which hampers construction of regulatory networks. Existing reference genomes capture only part of soybean’s genetic diversity [14]. In practice, different cultivars carry unique alleles and structural variants not represented in the reference. This leads to inconsistent network behavior across cultivars and environments [15]. In other words, a gene that is central to a stress-response network in one cultivar may behave differently in another (due to paralogs or copy-number differences) [16]. Such variability requires careful multi-genome analysis and may reduce a computational models’ predictive power [17].

Lupin similarly suffers from limited genomic resources, although recent efforts are improving the situation. Only in the past few years have high-quality reference genomes for several *Lupinus* species become available [18]. Hufnagel et al. [19] went on to assemble and publish the first comprehensive white lupin pangenome by sequencing 39 diverse accessions and defining core versus variable gene sets. Subsequent reviews have built on these foundations to outline emerging genomic and pre-breeding resources for the genus [3]. Nonetheless, thousands of lupin proteins still lack experimental validation. Most annotations rely on homology to other species, which can miss lupin-specific pathways (for example, unique alkaloid biosynthesis) [18]. As a result, network analyses might overlook important regulatory modules involved in stress tolerance or nutrient use. Functional studies in lupin are also difficult. Mutant libraries are scarce, and genetic transformation is notoriously inefficient. White lupin, in particular, often shows very low and genotype-dependent regeneration in tissue culture [20,21]. This poor regeneration and low transformation efficiency severely limit the creation of stable transgenic or gene-edited lupin lines [20]. Even the few available methods, such as *Agrobacterium rhizogenes*-mediated hairy root transformation remain labor-intensive and low-throughput [22].

While *M. truncatula* is recommended as a model legume due to its small diploid genome, nitrogen-fixing ability, and short development cycle [23], its resources are less extensive than those of other primary model systems, limiting the integration of its data into large-scale, comprehensive research projects. Recent studies on abiotic stress response have provided a better understanding of signaling pathways for drought, salt, and cold stress [24], and insight into nodulation responses to cold stress has been described [25]. These publications support research in other legumes by identifying common pathways and response signaling associated with agronomically important stressors.

### 2.2. Model Plants (Arabidopsis)

*Arabidopsis thaliana* benefits from a wealth of community-curated resources, including a high-quality reference genome [26] comprehensive functional annotation databases (covering gene ontology terms [27], documented regulatory interactions, metabolic pathway maps and protein–protein interactions [28], and well characterized mutant collections for experimental validation [29]. These assets simplify the incorporation of new omics datasets, but the sheer scale of information demands advanced computational approaches—such as network pruning algorithms, clustering techniques and machine learning inference to isolate the most relevant subnetworks for further study [7].

Applying insights from model systems to field crop species remains challenging. In Arabidopsis, omics studies typically occur under tightly controlled environmental conditions to enhance reliability of results. While this improves reproducibility, it limits the relevance to field environments where conditions vary and genotype by environment interaction can be abundant [8]. Gene networks associated with abiotic stress response under single-stress conditions may function differently when multiple stresses occur simultaneously, which can be common in agricultural settings [8]. For example, a network of genes induced by drought in isolation could be suppressed or altered if drought and heat occur together. Studies have shown that *Arabidopsis* plants under combined drought and heat stress have a transcriptomic profile that is not a simple sum of individual stress responses, but rather involves a distinct set of regulators [30]. Specifically, more than 770 unique transcripts are altered under combined stress, including those encoding heat shock proteins (HSPs, such as HSP70, HSP90, and HSP100), WRKY transcription factors, pathogenesis-related (PR) proteins, phenylalanine ammonia lyase (PAL), and ethylene response transcriptional co-activator (MBF1c) [30]. This highlights a general point: laboratory discoveries in *Arabidopsis* may not directly translate to crops facing complex field conditions. Additionally, Arabidopsis lacks key physiological traits important in legumes; it does not form symbiotic root nodules or fix nitrogen, nor does it produce critical seed compounds of interest in crops [31]. Networks derived from Arabidopsis may thus miss pathways or interactions critical for legumes like soybean and lupin. For instance, legume-specific genes required for nodulation (such as *Nodule Inception NIN* or *Calcium/Calmodulin-dependent protein kinase CCaMK*) simply have no orthologs in *Arabidopsis*, so any regulatory network involving those genes has to be studied in a legume context. Indeed, comparative genomics indicates a substantial divergence: *M. truncatula* shares only ~60% of its stress-responsive genes with *Arabidopsis*, meaning 40% have either diverged beyond recognition or are completely novel in legumes [32].

### 2.3. Cross-Species and Computational Barriers

A major barrier is ortholog divergence. Even when a soybean or lupin gene has an Arabidopsis counterpart, evolutionary drift can change its role. A transcription factor central in Arabidopsis might have a soybean homolog with different regulation or targets [33]. This divergence complicates cross species network analysis and may obscure truly conserved mechanisms. Existing translational genomics studies in legumes highlight this: only about 60% of abiotic-stress genes are conserved between *M. truncatula* and *A. thaliana* [32].

Computationally, most network tools were developed for well-studied organisms and may not suit so-called “orphan crops” (under-researched, non-model species) like lupin. Many annotation pipelines rely on similarity to reference genomes, which is problematic for lineage-specific genes. New tools are emerging: ExpressAnalyst [34] builds large ortholog databases to improve transcript assembly in non-model plants. Machine learning is increasingly being applied: deep learning can impute missing omics data [35] and improve annotations by recognizing gene features [36,37]. One promising avenue is transfer learning [38], where a model trained on a major crop or model plant is fine-tuned using a small amount of data from the orphan crop. For instance, a gene function prediction model built on *Arabidopsis* knowledge could be adjusted with known gene-function pairs from soybean, then applied to predict functions of uncharacterized lupin genes. MacNish, Danilevicz [39] discusses how conservation of genes between related species can be exploited in conjunction with machine learning to boost orphan crop improvement. Nevertheless, AI models trained on non-legume data may not capture legume-specific signals, so robust frameworks integrating diverse data types are needed. Validating AI-derived features and annotations poses its own challenges similar to functional validation of traditional annotations. Many legumes lack “gold-standard” reference datasets, so it is difficult to benchmark model predictions. Integrative approaches that combine data across species may help address this gap. For instance, Dick et al. [40] demonstrated that interaction data from *Arabidopsis thaliana* can be used to infer protein networks in *Glycine max*, using a cross-species prediction framework. Their approach highlights how leveraging model species data, in combination with machine learning, can extend network inference in non-model legumes where experimental resources remain limited.

### 2.4. Computational Network Analysis Tools (WGCNA and ARACNE)

WGCNA (Weighted Gene Co-expression Network Analysis) is a widely used correlation-based method for constructing gene co-expression networks in plants. WGCNA groups genes into modules based on their similar expression profiles across a sample set. It uses soft-thresholding of gene–gene correlation values to approximate scale-free network topology, emphasizing strong correlations while maintaining network connectivity [10]. This approach identifies modules of co-expressed genes and often designates a representative module eigengene (the first principal component of the module’s expression) that can be correlated with external traits or experimental conditions. Within each module, WGCNA pinpoints hub genes with high connectivity that may serve as key regulators. Because of its robust statistical framework and biologically meaningful groupings, WGCNA has been applied in numerous crop studies to discover gene networks underlying traits such as stress responses and development [10,12]. For example, He et al. (2021) used WGCNA to identify a module of anthocyanin biosynthesis genes in salvia with a central WRKY transcription factor as a hub, validating WGCNA’s ability to find functionally coherent gene sets [12].

ARACNE (Algorithm for the Reconstruction of Accurate Cellular Networks) is an information-theoretic algorithm used to infer gene regulatory networks. Unlike correlation-based methods, ARACNE evaluates pairwise mutual information between gene expression profiles to detect statistical dependencies (potential regulatory interactions) [41]. A key feature of ARACNE is the application of the Data Processing Inequality (DPI) to eliminate indirect connections, thereby pruning the network to retain only the most likely direct interactions [41]. This method is especially useful for constructing networks centered on transcription factors and their target genes, as it can capture non-linear and potentially causal gene–gene relationships that simple correlation might miss. ARACNE has been widely used in mammalian systems to reconstruct high-confidence regulatory networks, and plant researchers have begun to employ it for complex datasets as well. One limitation is that ARACNE’s computational complexity scales approximately quadratically with the number of genes, so it can be intensive on large transcriptome datasets [41]. Nevertheless, its ability to infer direct regulatory links makes it a valuable complement to co-expression approaches. In practice, using WGCNA and ARACNE in tandem can be powerful: WGCNA might first identify co-expression modules, and then ARACNE (or similar mutual-information methods) can be applied within modules or across key genes to infer direct regulatory relationships. By combining multiple network-inference techniques, researchers can cross-validate predicted interactions and build more robust models of plant molecular networks.

## 3. Bridging Discovery and Application

**Figure 1:**
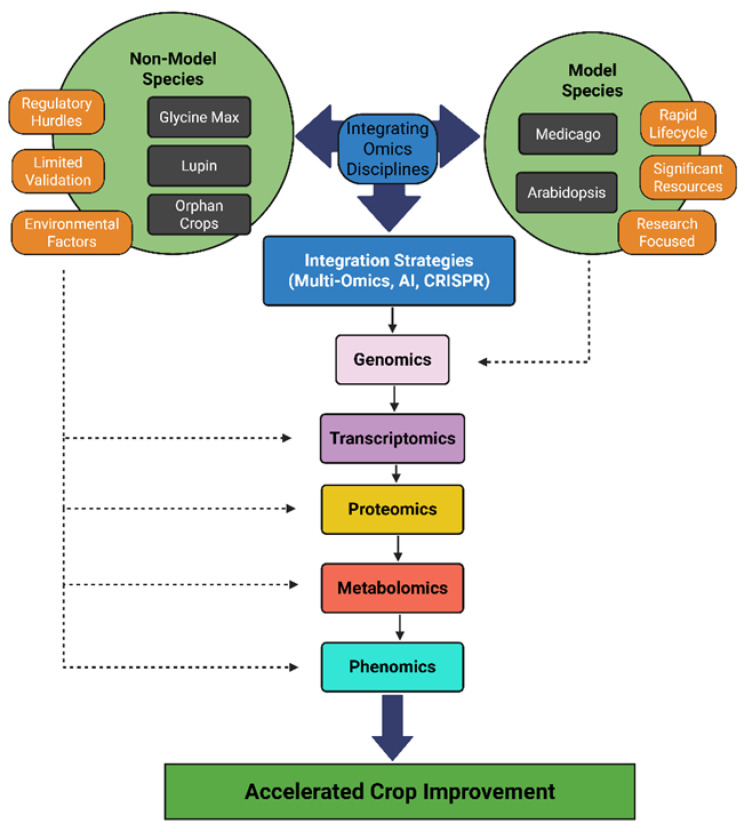
Conceptual overview showing how model and non-model legumes (soybean, lupin, *Medicago truncatula*, and Arabidopsis) inform integration strategies like multi-omics, AI, and CRISPR to connect omics layers with environment and support crop improvement. The schematic highlights cross-layer integration of data: genomic, transcriptomic, proteomic, metabolomic, and phenomic information (depicted as interconnected layers and arrows) are combined across these species. Computational tools such as AI facilitate the fusion of these omics layers, and validation methods like CRISPR link molecular findings to phenotypic outcomes. This cross-omics integration provides a holistic framework that connects gene networks to traits under real-world conditions, ultimately guiding breeding and biotechnology strategies for crop improvement. Created with BioRender.com.

Multi-omics integration constructs more comprehensive network models by layering genomics, transcriptomics, proteomics, phenomics and metabolomics data to capture the interplay between omics levels. Figure 1 provides a conceptual summary of how these integration strategies consolidate multi-layered omics data and environmental context to facilitate molecular network analysis and practical breeding outcomes. Combining metabolite profiles with gene expression can reveal regulatory modules controlling seed composition or stress responses in legumes. One strategy feeds multi-omics into predictive models: Wang et al. [42] developed deep neural network genomic prediction (DNNGP), using genomics and other omics data to improve agronomic trait prediction. They tested DNNGP on bread wheat lines with matched transcriptome and metabolome data and found it was 8–12% more accurate at predicting grain yield and drought tolerance than GBLUP (Genomic Best Linear Unbiased Prediction, a linear mixed-model using genomic markers), LightGBM (a fast gradient-boosting decision-tree algorithm), and SVR (Support Vector Regression, which fits a regression line within a specified error margin). By training on extensive panels of genotypes with known drought-tolerance or nutrient-use-efficiency phenotypes, DNNGP uncovers non-linear relationships between molecular features and field performance. This enables breeders to prioritize untested lines for traits like drought resilience or nutrient uptake based solely on their omics profiles—dramatically speeding up selection cycles and reducing dependence on expensive, time-consuming field trials.

Once candidate genes are identified, genome-editing tools enable functional validation and trait engineering. In soybean, CRISPR/Cas9 (Clustered Regularly Interspaced Short Palindromic Repeats and CRISPR-associated protein 9) editing of fatty-acid desaturase genes (*GmFAD2-1A/B*) doubled seed oleic acid content, yielding seed with high-oleic acid and improved oil stability [43]. In *M. truncatula*, efficient CRISPR/Cas9 mutagenesis of the phytoene desaturase gene, *MtPDS*, in hairy-root assays has demonstrated the system’s power for dissecting nodulation gene function [44]. Although still nascent in white lupin, CRISPR editing of the trehalase gene, Lalb_Chr05g0223881, in *Agrobacterium rhizogenes*-transformed hairy roots has successfully validated candidate genes linked to alkaloid content and drought tolerance [22]. These examples illustrate that gene editing can directly confirm the roles of predicted network hubs or modules (e.g., a metabolite transporter gene in a drought network can be knocked out to see if drought tolerance is reduced, thereby validating its importance). Notably, gene editing and transformation protocols are steadily improving for several orphan legumes. For example, researchers have adapted CRISPR/Cas9 for use in cowpea and pigeonpea by optimizing guide RNA delivery and using germline-specific promoters, achieving the first stable edited lines in those crops in recent years [45]. Moreover, even transient editing can yield insights: in chickpea, CRISPR has been delivered to protoplasts to knock out drought-responsive regulatory genes, demonstrating their roles in stress signaling without needing whole plant transformation [46]. Similarly, in cassava (a non-legume orphan crop), CRISPR knockout of the phytoene desaturase gene produces a visible albino phenotype, serving as a proof-of-concept for functional genomics in that species. These successes across diverse orphan species underscore that CRISPR/Cas9 systems can be effectively deployed beyond the usual model plants, given sufficient protocol optimization [46].

Genomic selection and predictive modeling use genome-wide markers in integration with omics techniques to predict breeding values and reduce the length of breeding pipelines. In soybean, genomic selection coupled with phenomics improves yield and quality predictions [47]; similar approaches are emerging in lupin [48]. Integrative breeding pipelines link genomic, transcriptomic, phenomic, and environmental databases with computational tools to guide selection. Integrating high-density genotype data, sensor-based phenomics, and weather into unified models can accelerate selection of stress-resilient varieties [49]. These approaches increase reproducibility, allow customization, and shorten breeding cycles.

## 4. Omics Integration Across Species

### 4.1. At the Genome Level

Genomics provides the foundation for any omics study. The soybean genome is well-characterized with relatively abundant genomic resources [2,14,36,50]. These rich datasets are readily accessible through community resources such as *SoyBase*—the USDA-ARS soybean genetics and genomics database that hosts reference assemblies, genetic maps, pan-genome variants and trait QTLs—alongside the *Legume Information System* [51], which offers comparative genomics tools across legume species [52]. Broad-scale plant genomics portals like *Ensembl Plants* and *Phytozome* provide genome browsers, standardized gene models and variant catalogs for *Glycine max*, while the *NCBI Genome Data Viewer* enables sequence retrieval, annotation browsing and BLAST-based searches [53,54,55]. By centralizing reference assemblies, gene annotations and variation datasets, these platforms not only streamline cross-species comparisons but also underpin downstream integration of transcriptomic, proteomic and metabolomic data. Long read sequencing efforts include the wild and cultivated soybean pan genome assembly [56] and the Pan Soy collection of over 200 accessions [57]. These studies have revealed extensive presence and absence variation and structural variants linked to key agronomic traits. In one study, genome-wide association studies (GWAS) pinpointed over 20 loci associated with seed composition traits, revealing candidate genes involved in lipid metabolism, carbohydrate partitioning, and transcriptional regulation [58]. Furthermore, translational genomics has begun to directly improve soybean crops: using CRISPR/Cas9 to knock out two fatty-acid desaturase genes (*GmFAD2-1A*/*1B*) elevated soybean seed oleic acid content by ~80%, demonstrating how genomic insights can be applied to enhance nutritional traits [43]. Such gene editing successes illustrate the power of soybean genomics for crop improvement.

Lupin historically lacked high-quality reference genomes until recent advances. Hufnagel et al. [18] produced a 451 Mb chromosome-level assembly for white lupin, alongside assemblies of a landrace and a wild relative revealing ~38,000 genes and providing insight into unique lupin adaptations. For example, the white lupin genome encodes the developmental machinery for cluster roots for phosphorus (P) uptake [18]. Early efforts by Hufnagel et al. [19] first established a white lupin pangenome through sequencing 39 diverse accessions, defining 32,068 core genes present in all lines and 14,822 variable genes that may underlie important trait variation. Notably, this pangenome analysis pinpointed the low-alkaloid *pauper* locus (conferring “sweet” seeds) as a key domestication gene in *L. albus* [59]. It also uncovered structural variants linked to phenology—for instance, indels in the promoter of *FT* (*Flowering locus T*) a flowering-time gene differentiate early- versus late-flowering lupins [60]. These genomic insights guide lupin pre-breeding by linking sequence variation to traits like seed quality and adaptation to environments. In parallel, metabolite QTL (mQTL) mapping is emerging as a powerful approach in lupins. For instance, Tosoroni et al. [3] associated variation in seed quinolizidine alkaloid (QA) profiles and galactooligosaccharide (GOS) content with specific genomic regions, laying the groundwork for marker-assisted selection of nutritional traits. This integration of metabolomic and genomic data represents a key step forward for trait-based breeding in lupins.

*M. truncatula* research provides insight into the genetic mechanisms associated with rhizobia interactions and nitrogen fixation—processes that, when optimized, can improve nitrogen-use efficiency, reduce reliance on synthetic fertilizers and enhance soil health for sustainable agriculture [61]. Since its genome was fully sequenced and annotated in 2011, *M. truncatula* has become a powerful model for legume omics analyses [62]. Building on foundational genomic resources, Curtin et al. [63] carried out functional validation of three key nodulation genes—demonstrating their essential roles in establishing legume–rhizobia symbiosis. More recently, GWAS in *M. truncatula* have identified additional loci linked to nodulation traits, further expanding our understanding of the genetic basis of symbiotic interactions [64]. Ref. [65] used GWAS to investigate symbiosis between *M. truncatula* and *Sinorhizobia* (*Ensifer*) *melitoti,* revealing that multi-strain inoculation may provide more opportunity for preferential nodulation. Beyond symbiosis-focused studies, broader genomics in *Medicago* are yielding translational insights. For example, a GWAS of 162 diverse *M. truncatula* lines identified dozens of loci controlling seed traits—79 QTNs (quantitative trait nucleotides) for seed size and 41 for seed composition (protein and sulfur content)—including one near a gene encoding an RNA-binding domain protein that appears to jointly regulate seed size and protein concentration [66]. Such findings in *M. truncatula* represent valuable resources that can be rapidly transferred to crop legumes like pea, common bean and soybean via syntenic mapping of these loci.

### 4.2. At the Transcriptome Level

Transcriptomics bridges the gap between static genomic information and dynamic cellular responses. In soybean, RNA-seq has been used extensively to profile gene expression during development and under stress [67,68,69]. For example, one study compared developing seeds of high-protein and high-oil soybean cultivars and found over 12,000 differentially expressed genes in lipid synthesis, amino acid metabolism, and hormone signaling pathways [58]. In high-protein cultivars, desiccation-tolerance and photomorphogenesis genes are activated sooner, and leaf senescence is postponed, potentially directing more nitrogen toward protein synthesis than in high-oil cultivars [58]. Additionally, transcriptomic profiling under abiotic stress has yielded insights into soybean’s drought-response networks [70]. Li et al. [71] exposed soybean plants to mild, moderate, and severe drought at flowering and found on the order of 2000–3600 genes induced under each stress level. Weighted gene co-expression network analysis highlighted drought-responsive transcription factors (TFs) in the WRKY, MYB, NAC, and bHLH families that act as regulators forming stress-response gene modules [71]. Together, these transcriptomic studies reveal the networks underlying soybean seed traits and drought adaptation.

Lupin transcriptomics has highlighted regulatory responses to nutrient stress and specialized metabolism. Under phosphorus deficiency, for example, RNA-seq of white lupin roots showed upregulation of genes involved in cluster-root formation, organic acid exudation, and high-affinity phosphorus transport [72,73]. A detailed study of individual white lupin cluster-rootlets found a coordinated switch in gene expression: as a rootlet transitions from growth to phosphorus-acquisition mode, growth-related genes are downregulated and phosphorus-uptake genes are turned on [73]. In the realm of secondary metabolism, transcriptome comparisons of bitter versus low-alkaloid (*pauper*) white lupin lines identified several quinolizidine alkaloid pathway genes that are downregulated or disrupted in the low-alkaloid lines [74]. This narrows the search for genes controlling alkaloid content. Similarly, in yellow lupin under drought stress, roots upregulate jasmonate biosynthesis and antioxidant enzyme genes, suggesting these pathways contribute to drought tolerance [75]. Overall, these studies show how lupin RNA-seq is uncovering stress-adaptive and metabolic gene networks.

*Medicago truncatula* benefits from rich transcriptomic resources. MtExpress, an RNA-seq atlas compiling data from thousands of experiments, is ideal for mining co-expression patterns or identifying stress-responsive genes [76]. The Affymetrix-based Medicago Gene Expression Atlas remains a foundational reference [77], while MtSSPdb (the *Medicago truncatula* Small Secreted Peptide database) catalogs ~4400 small secreted peptides with expression profiles, network partners, and mutant phenotypes [78]. Beyond these reference atlases, targeted transcriptomic studies are shedding light on how Medicago gene networks respond to stress. Chakraborty et al. [79] discovered that salt stress can *enhance* the expression of early nodulation genes in *M. truncatula*, and also induces a set of “stress-specific” rhizobium-responsive genes. This suggests crosstalk between abiotic stress and the symbiotic nodulation program. Emerging single-cell RNA-seq studies are further resolving the spatial complexity of gene expression during nodule development. In sum, the extensive transcriptomic data and analyses in *M. truncatula* make it a powerful model for uncovering legume gene networks and stress responses.

### 4.3. At the Proteome Level

Proteomics directly identifies and quantifies proteins and their post-translational modifications, providing insight into protein abundance, interaction dynamics, and regulatory states. In soybean, seed proteomics has been a major research focus for crop improvement, given the complex proteome of storage proteins, enzymes, and structural proteins governing oil and protein accumulation [80,81,82,83,84]. Detecting low-abundance regulatory proteins is challenging because they are masked by the highly abundant storage proteins, glycinin and β-conglycinin, which together constitute ~70% of seed protein [85]. Advanced mass spectrometry techniques have helped address this issue. Meng et al. [84] performed a deep proteomic analysis of developing soybean seeds from high-oil and high-protein cultivars. Using liquid chromatography–tandem mass spectrometry (LC–MS/MS) and label-free quantification, they identified thousands of seed proteins and pinpointed those that differed significantly between the two seed types. Functional enrichment and network analysis revealed clear distinctions: high-oil seeds showed elevated levels of enzymes for fatty acid biosynthesis and stress-defense proteins, whereas high-protein seeds had more proteins involved in nitrogen metabolism and antioxidant pathways. These proteomic differences help explain the metabolic basis for the inverse relationship between oil and protein content in soybean seeds [84]. As a complementary example, Islam et al. [86] used proteomic and metabolomic profiling to compare a high-protein soybean mutant to its wild-type parent. The mutant showed reduced ubiquitin–proteasome activity and increased heat shock proteins, suggesting that limiting protein degradation and enhancing protein folding contributes to higher seed protein levels. Proteomic studies have also revealed how soybean responds to abiotic stresses through coordinated changes in protein expression. Under drought conditions, tolerant genotypes upregulate proteins involved in sugar catabolism, antioxidant defense, and lipid metabolism, suggesting a strategy focused on energy mobilization and ROS detoxification rather than stress hormone signaling [87]. Similarly, in response to salt stress, soybean roots show reduced levels of vacuolar proton pumps (V-ATPase) and elevated antioxidant enzymes like peroxiredoxin. However, treatment with titanium oxide nanoparticles partially reversed these effects, maintaining ion transport and normalizing ROS levels [88]. Together, these studies highlight how stress tolerance in soybean is mediated by metabolic reprogramming, efficient ion regulation, and enhanced oxidative stress responses at the protein level. Proteomic investigations in lupin are essential for understanding stress tolerance mechanisms and seed biochemistry. A study on yellow lupin roots under drought stress found significant increases in lipoxygenase (LOX) enzymes catalyzing fatty acid oxidation to signaling molecules (jasmonates) and phospholipase D (PLD) involved in membrane lipid remodeling and stress signaling. In addition to stress responses, proteomic studies are illuminating lupin development and nutrition. For instance, a seed proteome survey of 46 narrow-leafed lupin genotypes revealed extensive natural variation in the major seed storage proteins (conglutins). Strikingly, some modern cultivars entirely lacked certain β-conglutin subfamilies—drastically reducing their total β-conglutin (a common allergen) content—while other conglutin families increased to compensate. This diversity suggests breeders could select low-allergen, high-protein lupin lines for food use (e.g., hypoallergenic flour) without sacrificing protein quality. Proteomics has also uncovered unexpected developmental insights: despite lupin seeds being protein-rich, germinating lupin seedlings do not primarily catabolize amino acids for energy, instead mobilizing stored lipids and converting them into sugars via gluconeogenesis [89]. Furthermore, proteomic analyses have identified bioactive lupin proteins such as γ-conglutin, a minor seed component (~5% of total protein) that remains intact during germination, which can lower blood glucose levels in animal and human studies [90]. This protein is now regarded as a potential antidiabetic nutraceutical, illustrating how proteomic discoveries link seed composition to valuable health traits [90]. These examples underscore how lupin proteomics is advancing our understanding of developmental processes and nutritional qualities, guiding improvements in crop breeding and food applications.

In *M. truncatula*, proteomic studies have investigated small, secreted peptides (SSPs) that contribute to many developmental stages, including nodulation. One study demonstrated the importance of 240 nutrient-responsive and 365 nodulation-responsive signaling-SSPs and highlighted the successful use of synthetic peptides in altering root growth and nodulation [91]. Building on these findings, a comprehensive database (MtSSPdb) was established to curate all known and predicted Medicago SSPs and their functions. This resource compiles *4439* SSP-encoding genes (including many small open reading frames previously overlooked in genome annotations) and integrates a broad gene expression atlas for *M. truncatula* [78]. Proteomic studies in *M. truncatula* are unveiling abiotic stress response pathways, often in combination with other omics. For instance, a recent quantitative proteome analysis tracked changes in the *M. truncatula* seed proteome during a controlled dehydration/rehydration seed priming regime [92] with dozens of proteins showing stress-responsive shifts in abundance. These results suggest that *M. truncatula* seeds, and by extension legume seeds in general, can be primed to “biochemically remember” stress—deploying a suite of repair enzymes and protective proteins that enhance seedling vigor under adverse conditions [92].

### 4.4. At the Metabolome Level

Metabolomics provides direct views of biochemical states by measuring small molecules produced through gene expression and enzyme activity. In soybean, this approach has been used to compare high-oil and low-oil lines, identifying lipid intermediates, glycerolipids, fatty acids, and sugars associated with seed composition [93]. Zhao et al. [94] profiled 30 soybean lines and identified 98 lipid-associated metabolites in developing seeds, with several showing strong correlations with seed oil content across genotypes. Metabolomic analyses are also elucidating soybean stress adaptations. For example, mass spectrometry imaging of soybean root nodules under drought and alkaline conditions showed significant shifts in nodule metabolism, including changes in the localization and abundance of isoflavones [95]. Such changes indicate that soybean nodules reallocate defensive metabolites under stress, potentially to protect the symbiosis. Similarly, targeted metabolite assays have found that drought-stressed soybean leaves accumulate osmolytes, like raffinose and proline, and antioxidant compounds, which correlates with improved dehydration tolerance [96].

Metabolomic analyses in lupin have focused on quinolizidine alkaloids (QAs)—bitter compounds that deter herbivores and are toxic to livestock and humans [97]. Modern liquid chromatography-mass spectrometry (LC-MS) methods detect and quantify major QAs rapidly, and recent surveys have leveraged this to screen germplasm [98]. Beyond alkaloids, lupin metabolomics explores other compounds like oligosaccharides and tocopherols, with recent analyses quantifying galactooligosaccharides (GOS) in different species and identifying genotypic variation [18]. These metabolite assays support breeding efforts by linking chemical phenotypes to genetic factors—for instance, screening of Mediterranean lupin landraces revealed that some carry the *pauper* marker allele for low-QA content, but metabolite measurements showed their actual seed alkaloid levels did not always align with the marker [59]. This underscores the importance of metabolomics in selecting truly “sweet” (low-alkaloid) lupin lines when marker predictions are uncertain.

In *M. truncatula* nodules subjected to salt stress, imaging mass spectrometry revealed increased arginine, indicating adjustments in nitrogen metabolism under saline conditions [99]. A Medicago Metabolite Atlas enables spatial visualization of compounds across defined root zones using integrated LC-MS and gas chromatography-mass spectrometry (GC-MS) datasets [100]. Moreover, integrative omics studies demonstrate the critical roles of certain metabolites in stress tolerance. For instance, a recent combined metabolomic and transcriptomic analysis found that flavonoid compounds are essential for *M. truncatula* to withstand the combined stress of cold and alkaline soils [101]. Integrative omics studies have also shown that drought stress triggers the accumulation of osmoprotective metabolites such as proline and *myo*-inositol, which support osmotic balance and protect against oxidative damage, highlighting the role of metabolites as central players in legume stress adaptation [102].

### 4.5. Phenomics and Computation

Phenomics comprises many scalable approaches ranging from automated imaging and sensor arrays to targeted assays to quantify plant traits, from morphological features to physiological parameters [103]. For soybean, three-dimensional depth cameras such as Intel’s RealSense D415 (Intel Corporation, Santa Clara, CA, USA)—integrated into handheld PlotCam platforms—have been validated under field conditions to monitor canopy height and architecture, correlating strongly (R^2^ = 0.95) with single-point LiDAR measurements [104]. Unmanned aerial vehicles fitted with hyperspectral sensors can capture canopy structure, plant height and leaf area index, and have been used to predict seed composition traits (e.g., protein and oil content) before harvest by linking reflectance signatures to biochemical assays [105]. Complementing aerial platforms, ground-based robots like the modular “ModagRobot,” equipped with red-green-blue (RGB) and depth sensors, traverse *G. max* rows to acquire RGB imagery and 3D point clouds, enabling high-resolution estimation of canopy cover, height and above-ground biomass (R^2^ ≈ 0.79 versus manual measures) [106].

In lupin, phenomics has been applied to root architecture studies, especially cluster roots in species like *L. albus* [19]. Chen et al. [107] used ROOTMAP, a 3D root architecture model, to simulate root growth responses in various conditions. Another application is disease resistance screening, with imaging-based quantification of lesion area to identify anthracnose-resistant white lupin germplasm using high throughput phenomics [108]. As phenomic techniques improve, researchers are beginning to apply them to non-model legumes like lupin in more comprehensive ways. For example, time-lapse imaging of lupin growth under nutrient stress has been used to characterize genotypic variation in root system plasticity [48], and UAV-based remote sensing is being tested for field-grown lupin to monitor canopy development and detect stress symptoms [109]. These efforts, although still in early stages, demonstrate the potential of phenomics to accelerate lupin improvement by objectively measuring traits that were previously labor-intensive to evaluate (e.g., root length, branching, or disease lesion progression).

In *M. truncatula*, high-throughput phenotyping has typically been used in controlled environments to capitalize on its short lifecycle and model status. Tran et al. [110] employed an automated imaging platform to monitor *M. truncatula* growth dynamics under different nutrient conditions, revealing nuanced effects of symbiosis on plant performance. Notably, even at high soil phosphorus, plants inoculated with arbuscular mycorrhizal fungi (AMF) showed a transient early growth advantage over non-mycorrhizal controls, although this benefit diminished at later stages [110]. By capturing subtle, quantitative differences in growth and physiology (e.g., shoot wilting rates, greenness retention, nodule formation) across many genotypes, these phenomic tools enable researchers to link phenotypic variation to underlying genes or alleles [110]. For example, Zhao et al. [94] combined transcriptomic and metabolomic profiling to correlate soybean seed oil content with co-expressed genes involved in lipid metabolism, identifying candidate regulators for this trait. Similarly, Tosoroni et al. [3] demonstrated that metabolite QTL mapping in white lupin can pinpoint loci underlying variation in seed alkaloid and oligosaccharide levels, illustrating how measurable phenotypic traits can be connected to specific gene networks through integrative analysis. Together, these studies illustrate how high-throughput phenotyping outputs, such as seed oil content or metabolite levels, can be systematically linked to gene regulatory modules or network hubs through integrative transcriptomic and QTL analyses, exemplifying the critical connection between phenomic traits and underlying molecular circuits. Other phenomics approaches, including hyperspectral imaging and metabolite profiling, have been used to predict salt tolerance before visible symptoms appear, while tools like the RhizoTube system enable non-invasive monitoring of root and shoot traits under drought stress [111]. As phenomics platforms are scaled to screen large mutant populations or natural variant collections of *M. truncatula*, they become powerful tools for forward genetics. In essence, high-throughput phenotyping in *M. truncatula* is enabling researchers to link phenotype to genotype with unprecedented precision [64]. By capturing subtle trait differences across hundreds of plants and correlating them with genetic data, scientists are rapidly identifying the genes and allelic variants that control complex traits like symbiotic effectiveness and stress resilience. This integrative strategy truly closes the loop between the species’ well-annotated genome and its rich phenotypic diversity, solidifying *M. truncatula*’s role as a bridge from model genomics to real-world crop improvement.

Machine learning has become indispensable for analyzing multi-omics data, powering tasks like variant effect prediction, network inference and integrated environment-aware models spanning genomics through phenomics [112]. Computational tools from machine learning frameworks to workflow managers and high-performance computing now power every layer of multi-omics research [113,114,115,116]. Advanced phenotyping methods pair machine learning with automated imaging to replace labor-intensive scoring with rapid, objective measurements. For instance, Naik et al. [117] captured simple RGB canopy images on a smartphone, then segmented the crop from the background and computed features like yellow-to-green pixel ratios, texture metrics and canopy cover. These automated imaging workflows produce massive amounts of time-series data that far exceed the capacity of manual analysis, making machine learning algorithms essential for efficient processing and interpretation [117]. By continuously monitoring plant development, extracting real time growth parameters and flagging stress symptoms remotely, these methods both save labor and greatly expand the phenotypic dataset available to breeders and researchers.

### 4.6. Emerging Technologies

Single-cell transcriptomics is a rapidly evolving tool with growing applications in plant research. Recent advances in spatial and multi-modal omics now enable simultaneous profiling of gene expression, chromatin accessibility, and metabolite signatures at cellular resolution, offering new insights into developmental and stress-related processes in plants [118]. Single-cell spatial enhanced resolution omics sequencing (scStereo-seq) has revealed gene expression gradients across different cell types in Arabidopsis leaves, elucidating developmental paths of vascular cells and guard cells [119]. Another powerful emerging technology is single-cell ATAC-seq (the assay for transposase-accessible chromatin with sequencing), which maps chromatin accessibility across individual plant cells. For example, Dorrity et al. [120] applied scATAC-seq to *Arabidopsis thaliana* roots, resolving cell-type-specific regulatory elements and integrating them with transcriptome data to infer developmental gene regulatory networks. Advanced spatial omics technologies which preserve cellular and molecular context within tissues have been used in soybean root system analyses and *M. truncatula* nodulation research, but the bulk of the publications using these new methods are in Arabidopsis [121]. These technological tools offer a new avenue of understanding for the complex systems involved in protein production and nodulation in legumes.

## 5. Cell Signaling Networks in Legume Responses

### 5.1. Signaling Pathways in Soybean

Understanding signaling pathways that regulate growth, stress response, and metabolism is essential for improving complex traits in legume crops. Omics technologies enable researchers to study these networks at a systems level, revealing how hormonal and environmental signals integrate to support development and adaptation under variable conditions.

Recent studies have delineated transcriptional networks regulating oil and protein biosynthesis in soybean seeds. For example, dozens of transcription factors and regulatory genes that respond to hormonal cues and nutrient levels to balance storage lipid versus protein synthesis. The transcription factor GmVOZ1A (a *Glycine max* VOZ-family protein containing a conserved C_2_H_2_-type zinc-finger domain) enhances seed fatty acid and oil accumulation by upregulating lipid biosynthetic genes, while loss-of-function mutations reduce seed oil content [122]. Hormonal pathways—particularly abscisic acid (ABA) and auxin—play integral roles in seed development and in modulating the protein–oil balance [123]. For example, dynamic changes in ABA levels correlate with shifts in fatty-acid biosynthesis during seed maturation [123], while auxin and ABA jointly influence key transcriptional programs that determine final seed size, yield and storage-compound allocation [124]. These studies highlight emerging omics approaches for mapping hormone-gene interactions used to develop targeted strategies for seed trait improvement.

### 5.2. Aluminum/Phosphorus Signaling in Lupin

Aluminum becomes soluble and toxic in acidic soils, where Al^3+^ ions bind to root cell walls and membranes, disrupting cell division and elongation at the root tip and impairing water and nutrient uptake [125]. White lupin counters this by activating hormone-mediated and calcium-dependent signaling cascades, along with specific transcription factors and microRNAs, to upregulate Al^3+^ transporters and detoxification enzymes that protect sensitive cells [125]. Coordinated regulation of gene expression and transporter activity enables lupin roots to detoxify Al^3+^ and protect sensitive cells [125]. Lupins also develop cluster roots in response to phosphorus or iron deficiency—a developmental program triggered by nutrient-stress signals that mobilizes otherwise inaccessible nutrients [73]. Transcriptomic and metabolomic profiling have pinpointed key regulators of cluster root development, including the transcription factor WUSCHEL-related homeobox 5 (WOX5), SCARECROW (SCR), a GRAS-family transcription factor essential for root radial patterning and stem cell maintenance, and the ROOT ONE (ROW1) protein [126]. Moreover, stress-responsive signaling pathways—including the ABA for drought response, jasmonate and ethylene pathways for defense—show variation between legume species [127]. Understanding these differences may help identify both conserved and species-specific regulatory components.

### 5.3. Nodulation and Stress Signals in M. truncatula

Spatial transcriptomics has demonstrated differential transcriptional regulation in *M. truncatula* root cells in response to *S. meliloti* inoculation [128]. These findings highlight the importance of cell-specific expression during rhizobia interaction in the early stages of nodule formation. Reactive oxygen species, particularly hydrogen peroxide, participate in signaling pathways associated with nodulation genes [129]. One of these genes, *M. truncatula* Serine/Threonine Protein Kinase 1 (*MtSPK1*), is induced by rhizobial lipo-chitooligosaccharide nodulation factors (LCOs) produced by *S. meliloti* [130]. Biotic stress from the root-knot nematode *Meloidogyne javanica* activates ethylene signaling pathways that antagonize perception of lipochitooligosaccharide (LCO) “nodulation signals” and subsequent nodulation gene expression, as revealed by transcriptomic profiling of infected roots [131]. Abiotic stressors similarly intersect with symbiotic signaling: salt stress induces transcriptional upregulation of *Nodulation Signaling Pathway 2* (*nsp2*), integrating osmotic-stress cues with the nodulation network to fine-tune target gene activation [79].

### 5.4. Applications of Signaling Networks for Crop Improvement

Molecular network analysis brings together different layers of omics data to uncover how genes and proteins work together to shape complex traits. Instead of looking at individual genes in isolation, this approach maps out the broader signaling networks that coordinate plant development and responses to the environment. Signaling networks inferred from omics pinpoint key regulatory hubs such as kinases, transcription factors, ubiquitin ligases and receptors that integrate environmental and developmental cues. Targeting these nodes can produce dramatic effects on complex traits. For instance, in understudied orphan crops such as sorghum and cassava, researchers have used genome editing tools (e.g., CRISPR-Cas9) to knock out or modify cytokinin receptor genes such as *SbHK1* (a cytokinin histidine kinase) and to upregulate or downregulate auxin response factors such as *MeARF2*, thereby reshaping plant architecture and enhancing drought resilience [47,132].

In soybean, a ubiquitin ligase that acts as a hub for seed size regulation in hormone signaling networks, has increased average seed weight and improved yield related traits [133]. In white lupin, phosphoproteomic and transcriptomic analyses revealed that the ABA-responsive transcription factor LuABI5 (a basic leucine zipper transcription factor that binds ABA-responsive elements) and the mitogen activated protein kinase LuMPK4 coordinate seed filling and heat stress responses [96]. By focusing on these central signaling components rather than peripheral network members, breeders and biotechnologists can harness system level leverage points and translate multi-omics insights into meaningful, system-wide improvements in legume performance.

Manipulating key components or hub genes within signaling pathways offers several distinct advantages. Hub regulators often control multiple downstream pathways, thereby offering a high potential for broad-spectrum resistance, an especially attractive trait for integrated pest and stress management [134]. For example, genome-wide and epistatic loci studies in soybean uncovered main and interacting loci conferring resistance to white mold (*Sclerotinia sclerotiorum*) across diverse environments [135]. Moreover, abiotic and biotic stress pathways often share components or interact through crosstalk mechanisms, meaning that targeting key regulatory hubs may simultaneously improve responses to both types of stress; however, because these pathways overlap, enhancing tolerance to one stress can sometimes compromise the response to another. Manipulating these nodes can also introduce multiple physiological barriers to pathogen infection or abiotic damage. This multi-layered defense architecture reduces the likelihood that pathogens or pests will evolve rapid countermeasures—a limitation commonly seen in transgenic and traditional breeding approaches relying on single-gene resistance [134]. For instance, *Phytophthora sojae* has overcome the effectiveness of multiple Rps genes introduced through traditional breeding [136], while soybean cyst nematode (SCN) populations have adapted to transgenic resistance derived from *Rhg1*-containing cultivars, reducing their long-term efficacy [137].

Phosphoproteomics and systems-level phospho-mapping will become vital tools in optimizing key signaling pathways. Phosphoproteomics refers to the large scale analysis of protein phosphorylation, the reversible addition of phosphate groups that dynamically regulates enzyme activity, protein–protein interactions and subcellular localization [138].

By decoding these cell signaling networks, researchers can devise strategies to rewire these pathways in crops—maintaining high yields by fine-tuning stress signaling or channeling nutrients more efficiently to seeds. As omics research continues to illuminate these complex networks, the ability to fine-tune crop physiology through targeted manipulation of signaling pathways becomes a realistic approach to enhancing crop performance [7,122].

## 6. Toward Sustainable Agriculture: Genetic Diversification

Omics advances in soybean and lupin carry broad significance for sustainable agriculture, food security, and biodiversity. Studying crops beyond traditional model species is essential when facing global challenges [139]. Soybean and lupin play pivotal roles in protein supply and ecological health, yet lupin remains under-studied. These under-studied crops may have unique adaptations making them suitable for specific conditions, but their breeding potential requires dedicated omics research. Each new gene or pathway uncovered through one omics approach enriches our comparative multi-omics toolkit and guides breeders to the most promising targets within and across species [140]. Equally valuable is the genetic diversity in wild and landrace legumes, as exemplified by the *Lupinus* genus, which spans hundreds of species adapted to acidic and nutrient poor soils as well as drought prone environments [3]. By characterizing and harnessing traits such as tolerance to high soil acidity and superior water use efficiency, breeders can introduce novel alleles into elite cultivars, expand cropping options, strengthen agro-ecosystem resilience and preserve dwindling crop genetic diversity. Future directions for orphan crop research will heavily emphasize translating knowledge from model and well-studied species those with fewer multi-omics and improvement resources. One aspect is network analysis and genome annotation: building high-quality reference genomes and functional annotations for orphan legumes like lupin, cowpea, bambara groundnut, etc., by leveraging homology and synteny with model legumes and Arabidopsis. Investments in omics infrastructure for underutilized legumes are especially important. This includes not just sequencing of diverse genotypes but developing databases, mutant populations, and protocols. The plant biology community has already seen how investing in *M. truncatula* resources paid dividends for legume biology. The future will likely bring pan-genomes for more orphan legumes; as Hu et al. [141] notes, applying pangenomics with advanced selection and genome editing can transform these neglected species into crops of broader significance.

### 6.1. Climate Resilience and Low-Input Agriculture

Soybean and lupin require minimal synthetic nitrogen fertilizer due to rhizobial symbiosis, reducing their overall carbon footprint. Their inclusion in crop rotations recycles soil nitrogen and breaks pest cycles, contributing to ecosystem health [142]. Legume rotation improves yield stability under stress by enhancing soil microbiomes and fertility [143]. In resource-scarce regions, developing high-yielding legume varieties that can thrive on marginal lands contributes to addressing protein malnutrition. Global initiatives like the African Orphan Crops Consortium are generating omics resources and training breeders to accelerate the enhancement of these orphan crops [144], effectively transferring omics-driven knowledge from models into tangible gains for climate-resilient agriculture. Early efforts like reviving European soybean cultivation through photoperiod adaptation genes [145] demonstrate how omics-identified alleles can adapt crops to new climates. By enhancing nitrogen use efficiency and stress tolerance, soybean and lupin are becoming pillars of climate-smart agriculture. As farm land becomes increasingly depreciated and climate change continues to alter the agricultural capacity of the planet, legumes benefit soil health, biodiversity, and ecosystem stability [142]. By rotating nitrogen-consuming crops such as corn and wheat with nitrogen-producing legumes, farmers can move towards a sustainable future without compromising income.

### 6.2. Limitations of AI and Multi-Omics Approaches

While the integration of AI and multi-omics is promising, several limitations temper their immediate application. High-dimensional omics datasets can suffer from noise and batch effects, and combining multiple data types often introduces data heterogeneity and missing values, which can lead to spurious correlations or overfit models if not carefully controlled [41]. AI models, such as deep learning networks, require large, representative training datasets, yet many non-model legumes lack sufficient high-quality data, limiting model performance and generalizability. Models trained on well-studied species or conditions may not capture legume-specific or field-specific signals, and the “black-box” nature of some AI algorithms makes it challenging to interpret biological meaning or gain regulatory insights from predictive models. Additionally, computational demands can be considerable: as noted, methods like ARACNE can become prohibitively slow with thousands of genes [41], and deep learning models often need specialized hardware. From a practical standpoint, the multidisciplinary expertise needed to integrate genomics, phenomics, and AI is still not common in many plant breeding programs. Recognizing these limitations is important, ongoing efforts in algorithm development (e.g., methods to handle missing data and improve model interpretability) and expansive data generation (e.g., establishing gold-standard reference datasets for orphan crops) are critical steps. By addressing issues of data quality, bias, and computational efficiency, researchers can ensure that AI-driven, multi-omics insights translate reliably into real-world improvements. In summary, although powerful, these tools must be applied with caution and rigorous validation to avoid misleading conclusions and to realize their full potential in legume crop improvement.

## 7. Conclusions

In summary, the use of omics technologies and network analysis in soybean, lupin, and *M. truncatula* is central to sustainable agriculture. Integrating diverse omics datasets enables identification of regulatory hubs and pathways that govern economically important crop attributes like seed composition, nutrient use efficiency, and stress tolerance. By utilizing diverse crop gene pools and enhancing omics resources of under-researched legumes we can unlock novel traits for climate-resilient cultivars. Continual advances in omics research are set to drive crop improvement, with targeted gene edits, predictive breeding models, and integrated pipelines paving the way for robust, profitable, high-yielding, and environmentally sustainable legume varieties. Investing in non-model plant research and in cross-species data integration is thus key for future crop resilience and food security. Looking ahead, major challenges remain in scaling network models across genotype–environment interactions and incorporating spatiotemporal expression patterns into predictive frameworks. Developing interoperable data standards and machine learning tools capable of integrating diverse omics and phenomics inputs will be essential to fully realize the translational potential of systems biology in legume breeding.

## Figures and Tables

**Table 1:** Comparative overview of model and non-model legumes relevant to molecular network analysis.

Species	Model Status	Genome Resources	Key Strengths	Key Challenges
*Arabidopsis thaliana*	Model	Extensive, curated	Comprehensive annotation, mutant libraries	Not a legume; lacks nodule formation
*Medicago truncatula*	Model legume	Moderate to high	Syntenic with legumes, N-fixation model	Fewer large-scale resources than Arabidopsis
*Glycine max* (soybean)	Non-model	Rich but complex	Genomic data, gene editing progress	Paleopolyploid, complicates annotation
*Lupinus albus*	Non-model	Emerging	Cluster root adaptation, nutrient-use traits	Poor transformation systems, limited mutants

## Data Availability

No new data were generated for this work.

## Abbreviations

The following abbreviations are used in this manuscript:

AI	Artificial Intelligence
CRISPR	Clustered Regularly Interspaced Short Palindromic Repeats
DEGs	Differentially Expressed Genes
GO	Gene Ontology
GWAS	Genome-Wide Association Study
KEGG	Kyoto Encyclopedia of Genes and Genomes
PCA	Principal Component Analysis
PPI	Protein–Protein Interaction
QTL	Quantitative Trait Loci
QTN	Quantitative trait nucleotide
R	R Statistical Software
RNA-seq	RNA sequencing
SNP	Single-Nucleotide Polymorphism
TF	Transcription Factor
WGCNA	Weighted Gene Co-expression Network Analysis
